# Kinesin Light Chain 1 Suppression Impairs Human Embryonic Stem Cell Neural Differentiation and Amyloid Precursor Protein Metabolism

**DOI:** 10.1371/journal.pone.0029755

**Published:** 2012-01-17

**Authors:** Rhiannon L. Killian, Jessica D. Flippin, Cheryl M. Herrera, Angels Almenar-Queralt, Lawrence S. B. Goldstein

**Affiliations:** 1 Department of Cellular and Molecular Medicine, University of California San Diego, La Jolla, California, United States of America; 2 Biomedical Sciences Graduate Program, University of California San Diego, La Jolla, California, United States of America; 3 Howard Hughes Medical Institute, University of California San Diego, La Jolla, California, United States of America; University of Nebraska Medical Center, United States of America

## Abstract

The etiology of sporadic Alzheimer disease (AD) is largely unknown, although evidence implicates the pathological hallmark molecules amyloid beta (Aβ) and phosphorylated Tau. Work in animal models suggests that altered axonal transport caused by Kinesin-1 dysfunction perturbs levels of both Aβ and phosphorylated Tau in neural tissues, but the relevance of Kinesin-1 dependent functions to the human disease is unknown. To begin to address this issue, we generated human embryonic stem cells (hESC) expressing reduced levels of the kinesin light chain 1 (KLC1) Kinesin-1 subunit to use as a source of human neural cultures. Despite reduction of KLC1, undifferentiated hESC exhibited apparently normal colony morphology and pluripotency marker expression. Differentiated neural cultures derived from KLC1-suppressed hESC contained neural rosettes but further differentiation revealed obvious morphological changes along with reduced levels of microtubule-associated neural proteins, including Tau and less secreted Aβ, supporting the previously established connection between KLC1, Tau and Aβ. Intriguingly, KLC1-suppressed neural precursors (NPs), isolated using a cell surface marker signature known to identify cells that give rise to neurons and glia, unlike control cells, failed to proliferate. We suggest that KLC1 is required for normal human neural differentiation, ensuring proper metabolism of AD-associated molecules APP and Tau and for proliferation of NPs. Because impaired APP metabolism is linked to AD, this human cell culture model system will not only be a useful tool for understanding the role of KLC1 in regulating the production, transport and turnover of APP and Tau in neurons, but also in defining the essential function(s) of KLC1 in NPs and their progeny. This knowledge should have important implications for human neurodevelopmental and neurodegenerative diseases.

## Introduction

Normal cellular organization and function requires intracellular transport driven by molecular motors. Kinesin-1 is a microtubule-based motor, moving cargos towards the plus end of microtubules [1], [2] in neurons and in other cell types. Kinesin-1 is composed of a pair of heavy chains (KHCs), which use ATP hydrolysis to power movement on microtubules, and a pair of light chains (KLCs), which regulate KHC activity and mediate cargo attachment [3]. Mammalian Kinesin-1 is assembled from three KHC gene products - Kinesin-1A, -1B or 1C (formerly KIF5A, KIF5B or KIF5C, respectively) and four KLCs (KLC1, KLC2, KLC3, or KLC4) [4]–[8].

In mice, Kinesin-1 subunits have tissue specific expression patterns: Kinesin-1B and KLC2 are ubiquitously expressed while Kinesin-1A, Kinesin-1C and KLC1 are enriched in neural tissue [7]–[10]. As revealed by animal studies in which various subunits are altered, Kinesin-1 plays important roles in the nervous system. For instance, Kinesin-1C and KLC1 mutant mice exhibit reductions in brain size and/or white matter tracts [9], [11] and cultured primary neurons with reduced Kinesin-1B, Kinesin-1C or KLC1 subunits have shorter neurites [12]–[14]. Kinesin-1A, Kinesin-1C and KLC1 mouse mutants exhibit loss of specific neuron populations [9], [11], [15]. Further, mutations in fruit fly KHC, KLC or mouse Kinesin-1A or KLC1 lead to axonal transport defects [15]–[18]. These observations suggest that specific Kinesin-1 subunits may have multiple functions in the nervous system.

Kinesin-1 is a major anterograde motor driving transport into the axons of neurons and faulty axonal transport may contribute to neurodegenerative diseases [19]. Alzheimer's disease (AD) is characterized pathologically by the presence of brain amyloid plaques and neurofibrillary tangles, the principle components of which are the amyloid precursor protein (APP) proteolytic cleavage product Aβ and the axonal microtubule associated protein Tau. APP is transported to synapses in a Kinesin-1 dependent manner and associates closely with KLC [20]–[23]. Tau also interacts with KLC1 and may be transported in the axon by Kinesin-1 [24]. KLC1 mutant mice have hyperphophorylated Tau [11], [25] and APP transgenic mice with reduced KLC1 function exhibit earlier and accentuated brain amyloid plaques, thought to be caused by abnormal APP transport and/or cleavage [26]. Together these data lead us to suggest that KLC1 can modulate APP and Tau function but this is challenging to test in human neurons.

Progress in understanding human development and disease is limited by a lack of appropriate human model systems. While model organisms and human immortalized cells will continue to provide useful information, species or cell type differences restrict their utility. Human embryonic stem cells (hESC) [27] offer important benefits for modeling human development and disease [28], [29]. Not only are hESC a potential source of all human cell types, including neural precursors (NPs) and neurons, but they also proliferate indefinitely in culture, are genetically malleable, and express proteins under endogenous transcriptional, translational and post-translational control. Thus, we used hESC as a source of neural cells to probe human neural development and possible roles of transport in neurodegenerative disease pathways in AD. In this study we engineered hESC to express reduced levels of KLC1 using small hairpin RNA (shRNA) targeted to KLC1 and examined whether suppression of endogenous KLC1 impairs human neural differentiation or endogenous human APP metabolism, which is implicated in AD.

## Materials and Methods

### Cell culture and subcloning of undifferentiated Hues9 hESC lines

DNA oligonucleotides targeting KLC1 exon 2 (Forward 5′-TGTAATTTGGTGGAGGAGAATTCAAGAGATTCTCCTCCACCAAATTACTTTTTTC-3′ and Reverse 5′-TCGAGAAAAAAGTAATTTGGTGGAGGAGAATCTCTTGAATTCTCCTCCACCAAATTACA -3′were subcloned as described [30] into pSicoR, or a modified pSico derived plasmid lacking the CMV-GFP cassette (Figure S1A). Vesicular stomatitis virus G protein pseudotyped lentivirus was prepared at the University of California, San Diego Vector Development lab to a titer of 10^8^ colony forming units/ml.

Undifferentiated Hues9 hESC lines were maintained as described [31]. To derive Hues9 lines with reduced KLC1 Hues9 were exposed to lentivirus encoding KLC1 shRNA and plated at limiting dilution. Single hESC colonies were expanded and viral insertion confirmed by PCR. Cells were karyotyped by Cell Line Genetics (Madison, WI) (Figure S1B).

### Neural differentiation

For differentiating hESC using the embryoid body (EB) method [32], confluent cultures of *shKLC1-1*, *shKLC1-2* and uninfected parental control hESC were dispase treated (BD; 1∶50 in hESC media), scraped and transferred to bacteriological grade petri dishes in hESC media lacking FGF2, but containing 1 mM Rho-associated protein kinase inhibitor (Y27632 or trans-4-[(1R)-1-Aminoethyl]-N-4-pyridinylcyclohexanecar boxamide dihydrochloride; Calbiochem). On day five, EBs were plated onto matrigel (BD) treated tissue culture plate in insulin, transferrin and selenium (ITS) media (Dulbecco's minimum essential medium (DMEM)/F12, penicillin streptomycin (both from Invitrogen) and ITS supplement (Sigma)). Medium was replenished every other day thereafter.

For PA6 feeder differentiation, mouse PA6 stromal cells [33] were cocultured with *shKLC1-1*, *shKLC1-2* and unmodified parental control hESC as described [34]. In brief, PA6 feeder cells were plated at 6400 cells/cm^2^ in growth media (high glucose DMEM, FBS, glutamine, penicillin and streptomycin). The following day hESC were seeded onto the PA6 feeder at a density of 13 cells/cm^2^ (for control and *shKLC1-1*) or 50 cells/cm^2^ (*shKLC1-2*) in PA6 differentiation media. The medium was exchanged on day 6 and every other day thereafter.

### Neural precursor culture and viral transduction

Sorted Hues9-derived NPs were grown on polyornithine and laminin coated plates in NP media. Medium was exchanged every other day and cultures were split every 3–4 days. When nearly confluent, Hues9 derived NPs were transduced with virus containing CMV-GFP reporter cassette and U6-shKLC1 or U6-shLUC control shRNA and centrifuged at 800× *g* for 45 min at room temperature. Following expansion for 1–2 passages, cells were subjected to fluorescence activated cell sorting to enrich for GFP+ cells and cultured (Figure S5).

To differentiate NPs to neurons, NPs were plated on polyornithine and laminin coated plates in NP media and grown until they reached 70% confluence. FGF was removed and NP media supplemented with 20 ng/ml BDNF (Peprotech), 20 ng/ml GDNF (Peprotech) and 0.5 mM dibutyryl cAMP (N6,2′-O-Dibutyryladenosine 3′,5′-cyclic monophosphate sodium salt; Sigma). Medium was exchanged every 2–3 days.

### Brightfield imaging of cultures

To track morphology of the cells, cultures were imaged using a Nikon Eclipse TS100 and a Sony Power Shot G3. The camera was set to landscape mode, manual focus and images were collected with 5.7× zoom. A micrometer was used to calibrate the images.

### Flow cytometry

Pluripotency of undifferentiated Hues9 lines was assessed by flow cytometric analysis of Oct3/4 and TRA-1-81. Cells were dissociated with accutase (Invitrogen), fixed in 4% paraformaldehyde, permeabilized, incubated with primary antibodies (direct conjugates from BD) and suspended to 1–2×10^6^ cells/ml in sort buffer (1% FBS, 2.5 mM EDTA, 25 mM HEPES in PBS).

Differentiated hESC cell derived neural induction cultures were rinsed with PBS, dissociated with a 1∶1 mixture of accutase and accumax, filtered through 100 um mesh to remove cell clumps and an aliquot counted on a hemocytometer. Antibodies (BD) were added to a final cell concentration of 1–5×10^7^cells/ml. Labeled cells were sorted at 2.5–5.0×10^6^ cells/ml in neural precursor (NP) sort media (NP media - DMEM/F12, Glutamax, B27, N2, penicillin/streptomycin (all from Invitrogen) and 20 ng/ml bFGF (Peprotech) - supplemented with 10% FBS and 0.5 mM EDTA). To estimate the fraction of dead cells, separate aliquots of cells were stained with 750 nM propidium iodide, a membrane impermeable DNA binding dye, and analyzed by flow cytometry. Two to three percent of the cells were propidium iodide positive regardless of the cell line or neural induction method employed.

Cells were analyzed or sorted on a BD Biosciences FACSAria cytometer using a 100 micron diameter ceramic nozzle and 20 pounds per square inch sheath pressure. Single stained cells or CompBeads (BD) and FACSDiva software were used to calculate compensation values prior to analysis. Doublets were excluded from analysis with gates on forward and side scatter bivariate plots of pulse height relative to width (Figure S1C and Figure S2B). Antibody positivity (Figure S1D and Figure S2B) was defined by comparison to unstained controls. Analysis was conducted offline using FCS Express (De Novo Software).

### Western blot analysis of protein levels

Tissue culture lysates were prepared using PARIS kit (Ambion) buffer supplemented with protease (cocktail set I, Calbiochem) and phosphatase (Halt, Pierce) inhibitors. The BCA assay (Pierce) was used to estimate the protein content. Equal protein amounts were separated in MES buffer alongside Novex Sharp prestained markers (Invitrogen) on NUPAGE 4–12% acrylamide precast gels (Invitrogen) and then transferred to nitrocellulose (0.2 or 0.45 µm pore size Immobilon Millipore). Membranes were blocked in 5% BSA in tris buffered saline with 0.1% Tween-20. Primary antibodies (KLC1 H75 1∶500 Santa Cruz Biotechnology; Kinesin-1C 1∶500 C.H. Xia (unpublished); Actin C4 1∶100,000 Millipore; GFAP 1∶500 Dako; NSE 1∶1000 Millipore; MAP2 AP20 1∶1000; α-Tubulin DM1A 1∶50,000 Sigma; β-III-Tubulin TUJ1 1∶1000 Covance; phosphorylated neurofilament – heavy and medium chains (pNF-H and pNF-M, repectively) SMI31 1∶1000 Covance; Tau Tau-46.1 1∶500 Millipore; pTau PHF1 1∶500 Peter Davies; APP N terminus LN27 1∶250 Invitrogen; APP C terminus 1∶250 Zymed/Invitrogen; 1∶250 SOD1 Santa Cruz Biotechnology; GAPDH, 1∶3000 Ambion; Sox1 N23-844 1∶1000, BD; Nestin 1∶1000 Millipore; GFP 11E5 1∶1000 Invitrogen) were prepared in 5% BSA. Fluorescent secondary antibodies (LiCor) were diluted 1∶6000–15,000. LiCor Odyssey infrared imager was used to measure pixel intensities of bands at detector settings set the maximum or one half unit below saturation. For each protein band, background subtracted integrated intensity values were calculated using the Odyssey software. Since absolute integrated intensity values vary for the same samples on different blots, samples within a blot were plotted relative to control and these normalized values were used to average replicates from separate blots. Linearity of antibody response was verified over the range of 1–10 µg. To show protein bands in the conventional manner with dark bands on a light background, grayscale images were inverted in the figures.

### Immunofluorescence

Undifferentiated, *shKLC1-1*, *shKLC1-2* and uninfected parental control were characterized by immunofluorescence for KLC1, Oct-4 and TRA-1–81. Cells were fixed in 4% paraformaldehyde/0.12 M sucrose. For intracellular staining of Oct-4, KLC1, Sox1 and Nestin, cells were permeabilized with 0.1% Triton X-100, blocked with 10% FBS in PBS, and incubated with KLC1 (H75 rabbit IgG, 1∶400, Santa Cruz Biotechnology) Oct-4 (C10 mouse IgG2b 1∶300, Santa Cruz Biotechnology), Sox1 (mouse IgG 1∶1000, BD Biosciences) and Nestin (rabbit IgG, 1∶2000, Millipore) primary antibodies. Secondary antibodies Alexa Fluor 568 goat anti-rabbit IgG (H+L) or Alexa Fluor 568 goat anti-mouse IgG (H+L) antibodies (both from Invitrogen) were used at 1∶750 with 0.1 mg/ml 4′,6′-diamidino-2-phenylindole (DAPI; Sigma) nuclei stain prior to mounting on slides with Prolong Gold antifade reagent (Invitrogen). Specificity of secondary antibody staining was verified using secondary only controls. For cell surface TRA-1-81 staining cells were fixed and blocked as above and Alexa Fluor 647 conjugated TRA-1-81 primary antibody (BD Biosciences) was used at 1∶10. Fluorescence images were collected using a Zeiss Axioplan microscope equipped with a Zeiss Plan Neofluor 20×/0.50 NA objective, Texas Red and Cy5 filters, a CoolSNAPcf camera (Roper Scientific) and MetaMorph (Molecular Devices) software.

To quantify the percent of Sox1 or Nestin-positive cells in sorted NP cultures, the Image-based Tool for Counting Nuclei (University of California – Santa Barbara Center for Bio-Image Informatics) plug-in of ImageJ (NIH) was used to count the number of Sox1, Nestin and DAPI-positive cells per image. Care was taken to ensure that the best possible input parameters were used so that the algorithm identified cells that would also be manually judged positive. The proportion of Sox1 and Nestin-positive nuclei was calculated from a total of 932 DAPI nuclei. Technical replicates were used to calculate the standard error of the mean.

### Aβ and soluble APP (sAPP) quantification

To measure secreted Aβ and sAPP from hESC derived neural cultures, cultures were differentiated as described and the media changed completely 24 hours before harvest. Media was collected and supplemented with protease inhibitors. Cells were scraped in homogenization buffer (20 mM Tris pH 7.4, 1 mM EDTA, 1 mM EGTA, 250 mM sucrose, protease and phosphatase inhibitors) and homogenized. Aβ-40, -42 and -38 and sAPP-α and -β were quantified in media or lysate solubilized in 1% Triton X-100 using multiplex Meso Scale Discovery electrochemiluminescence immunoassays with human specific antibodies according to kit instructions. The data were normalized to the total protein in the lysate. Since the fraction of neurons may differ between cultures, we normalized the APP cleavage product to neuron-specific enolase (NSE) lysate levels.

## Results

### KLC1 suppression does not alter hESC morphology or pluripotency marker expression

**Figure 1:**
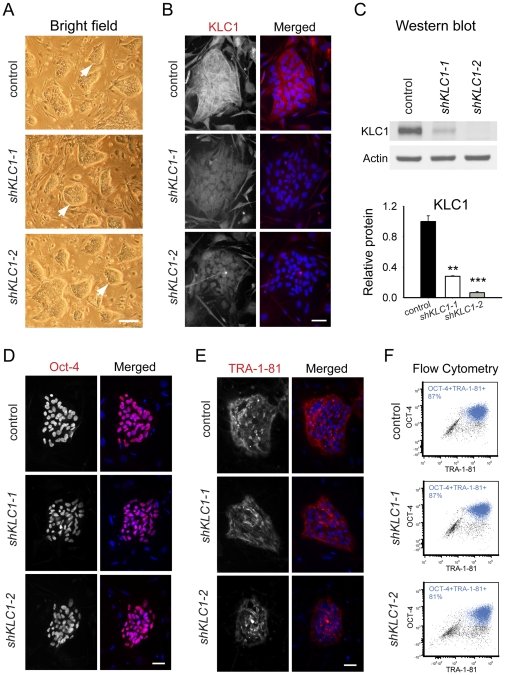
Undifferentiated KLC1-suppressed hESC exhibit normal morphology, pluripotency marker expression and karyotypes. (A) Representative images of control, *shKLC1-1* and *shKLC1-2* undifferentiated hESC cultures showing bordered colony morphology typical of pluripotent cells (arrows). Scale bar 200 micrometers. (B) Immunofluorescence staining of KLC1 in undifferentiated hESC control, *shKLC1-1* and *shKLC1-2* colonies. Merged images show overlay of KLC1 (red) and DAPI-stained nuclei (blue). Scale bar 50 micrometers. (C) Equal protein from undifferentiated control, *shKLC1-1* and *shKLC-2* culture lysates were analyzed by Western blot for KLC1 and Actin. Bar graph shows Actin normalized KLC1 levels relative to control. n = 3; **p<0.01, ***p<0.001 by 2-tailed t-test compared to control. (D–E) Immunofluorescence imagesof undifferentiated control, *shKLC1-1* and *shKLC1-2* cultures for pluripotency markers Oct-4 (D) and TRA-1-81 (E). Merged images show overlay of Oct-4 (D; red) or TRA-1-81 (E; red) and DAPI-stained nuclei (blue). Scale bar 50 micrometers. (F) Bivariate plots show distribution of cells in control, *shKLC1-1* and *shKLC1-2* undifferentiated cultures Oct-4+TRA-1-81+ (in blue). Data is representative of three experiments.

To obtain undifferentiated KLC1-suppressed hESC, we transduced Hues9 hESCs [31] with two different lentivirus [35] coding for a KLC1-specific shRNA (referred to here as *shKLC1-1* or *shKLC1-2*; S1A) [30]. Using limiting dilution plating single colonies were obtained, expanded, screened by PCR for the viral insertion and one clone of each selected for further analysis. Cell clusters from these lines exhibited well-bordered colony morphology typical of pluripotent stem cells (Figure 1A, arrows). Reduction of KLC1 protein levels in undifferentiated *shKLC1-1* and *shKLC1-2* compared to control hESC was confirmed by both immunofluorescence (Figure 1B) and immunoblot (Figure 1C).

Normal karypotypes suggest neither the subcloning process nor KLC1 reduction caused gross cytogenetic instability (Figure S1B). Fluorescence micrographs of Octamer-4 (Oct-4) and Tumor Rejection Antigen 1–81(TRA-1-81) revealed similar cellular distributions of Oct-4 in the nucleus (Figure 1D) and TRA-1-81 on the cell surface (Figure 1E) of control and KLC1 depleted cells suggesting depletion of KLC1 has no obvious effect on localization of these two pluripotency markers. Using flow cytometry, we found similar proportions of *shKLC1-1*, *shKLC1-2* and control cells expressing both Oct4 and TRA-1-81 (Figure 1F). From these data we suggest that undifferentiated pluripotent cells with 70–80% reduced KLC1 exhibit apparently normal colony morphology, karypotypes and pluripotency marker expression.

### KLC1-suppressed human neural cells exhibit shorter neurites

**Figure 2:**
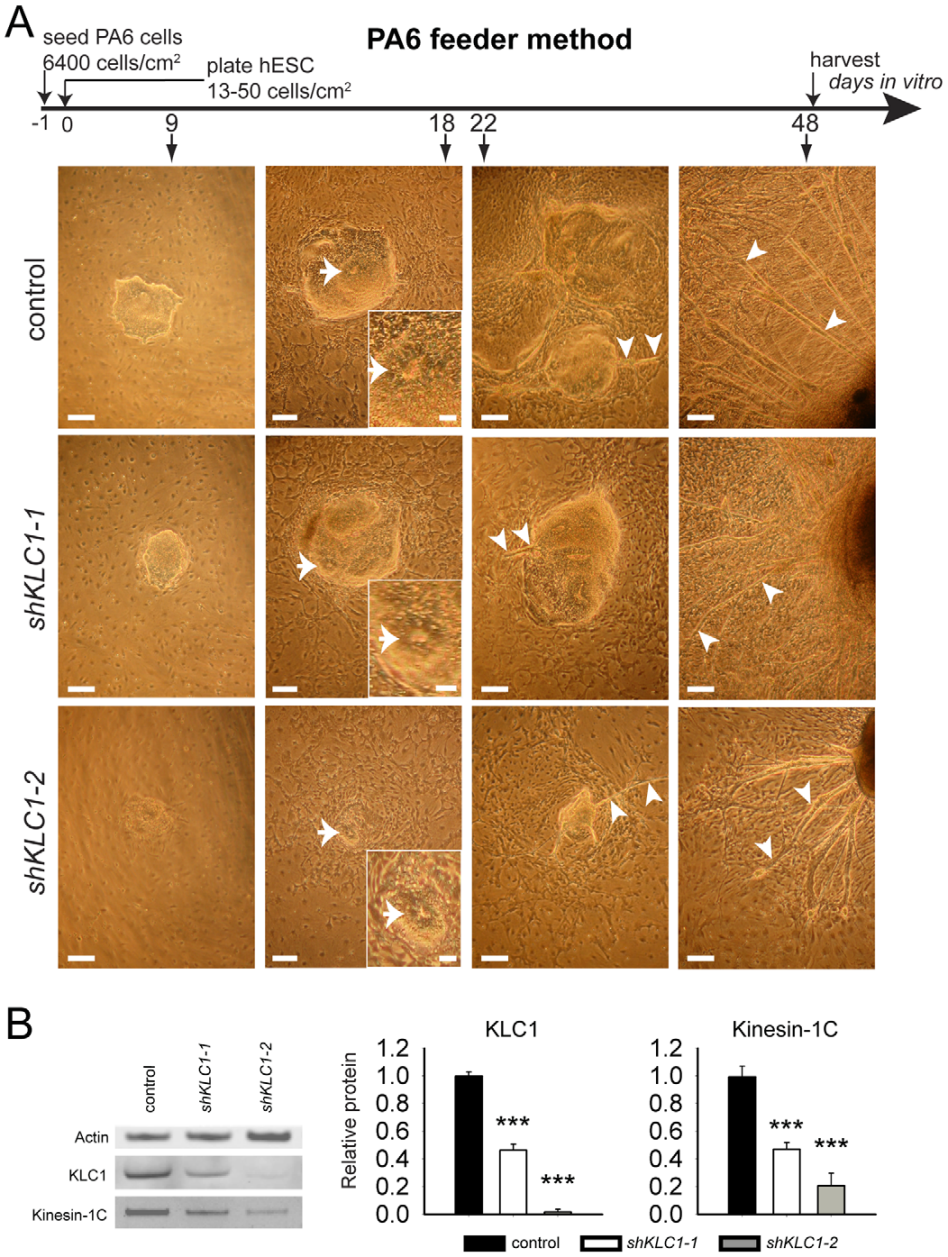
KLC1 and Kinesin-1C subunits are reduced in neural cultures derived from KLC1-suppressed hESC. (A) Control, *shKLC1-1* and *shKLC1-2* hESC were differentiated for seven weeks using the PA6 feeder method. Representative bright field images of control, *shKLC1-1* and *shKLC1-2* PA6 feeder cocultures collected at nine, eighteen, twenty-two and forty-eight days after plating. Arrows point to rosettes. Insets show close-ups of indicated rosettes. Arrowheads denote axon-like projections emanating from hESC derived cell clusters. Scale bars: 200 µm for main images, 50 µm for insets. (B) PA6 neural differentiation cultures were harvested after seven weeks *in vitro* and equal protein from control, *shKLC1-1* and *shKLC1-2* cultures analyzed by Western blotting for KLC1, Kinesin-1C and Actin. Bar graphs show relative quantification of KLC1 and Kinesin-1C levels relative to Actin. Based on n = 7 control and *shKLC1-1*; n = 3 *shKLC1-2*, ***p<0.001 by two-tailed Student's t-test compared to control.

Several lines of evidence suggest that neurons with impaired Kinesin-1 subunits may be smaller. KLC1 mutant mice have reduced white matter in the brain and spinal cord [11], Kinesin-1C mutant mice have smaller brains [9] and various dysfunctional Kinesin-1 subunits leads to reduced neurite lengths [12]–[14], [36], [37]. We tested whether human neurons with depleted KLC1 were similarly impaired. The PA6 feeder method has been shown to generate mature neuronal cultures [34], so we used it to generate and compare control and KLC1-suppressed mature neural cultures by differentiating hESC *in vitro* for seven weeks. We examined the morphology over time of control, *shKLC1-1* and *shKLC1-2* PA6 feeder differentiation cultures using bright field imaging. By *in vitro* differentiation day nine, hESC derived cell clusters peppered the feeder cell monolayer (Figure 2A, left panels). Since human neural differentiation from hESC generally follows human embryonic developmental principles [38], we observed differentiation cultures eighteen days from the undifferentiated hESC state, during the developmental equivalent of the neural tube stage. At this stage, control, *shKLC1-1* and *shKLC1-2* hESC derived day cultures contained ‘rosette’ cell cluster structures (Figure 2A arrows and insets) which resemble the neural tube and are typically found in neural induction cultures [38], suggesting the cultures may contain NP cells. By differentiation day twenty-two these hESC-derived cell clusters had sprouted axon-like projections, which persisted at least until differentiation week seven (compare right-most two panels of Figure 2A). While all lines followed this differentiation progression, *shKLC1-1* and especially *shKLC1-2* appeared to exhibit reduced cell cluster size and overall cell density over the course of the differentiation (Figure 2A and Figure S2) and less extensive and shorter projections in polarized cells at the time of harvest (Figure 2A, arrows in right-most panel and Figure S2). We wondered if the severity of phenotype correlated with KLC1 knockdown efficiency. Therefore, we used Western blotting to assess KLC1 protein levels in culture lysates from differentiated control, *shKLC1-1*, and *shKLC1-2 hESC*. We observed that KLC1 levels in *shKLC1-1* and *shKLC1-2* were reduced to 46% and 2% of control levels, respectively (Figure 2B), suggesting that knockdown is maintained for at least seven weeks of PA6 feeder differentiation. Previous experiments in *D. melanogaster* and *M. musculus* suggested that genetic reductions in KLC can lead to reduction in KHC and vice versa (unpublished data). We extended these observations to human neural cultures and found reduced Kinesin-1C in KLC1-suppressed differentiation cultures - to 47% and 20% of control (Figure 2B). These data indicated to us that reduced levels of KLC1 and/or Kinesin-1C may impair neural differentiation in a dose dependent fashion.

### KLC1-suppressed human neural cells have reduced levels of microtubule-associated proteins and altered APP metabolism

**Figure 3:**
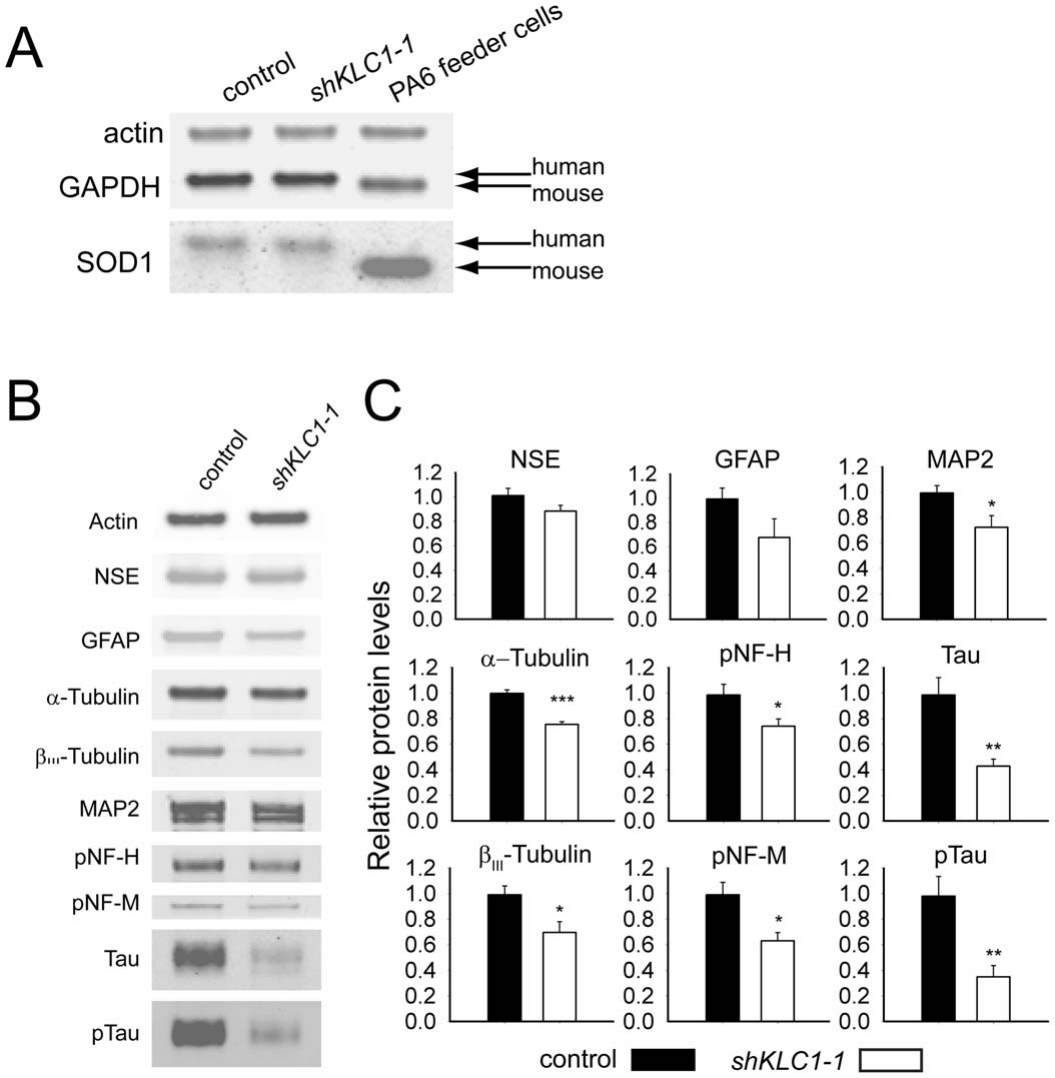
Neural cultures derived from KLC1-suppressed hESC have reduced neural microtubule-associated markers. (A) Cultures were harvested at seven weeks *in vitro* and equal protein from mouse PA6 feeders cultured with control, *shKLC1-1* or no hESC (PA6 feeder cells lane) were analyzed by Western blotting for actin, GAPDH and SOD1. Note that unlike Actin, mouse and human GAPDH and SOD1 have different electrophoretic mobilities (arrows). (B–C) Control or *shKLC1-1* hESC were cultured for seven weeks with PA6 feeder cells and then harvested. Equal protein from control and *shKLC1-1* cultures was analyzed by Western blotting. (B) Representative immunoblots of Actin, NSE, GFAP, α-Tubulin, β-III-Tubulin, MAP2, pNF-H, pNF-M, Tau and pTau. (C) Quantification of protein levels relative to control and normalized to Actin. Based on n = 6 wells each *p<0.05, **p<0.01, ***p<0.001 by 2 tailed t-test compared to control.

To discover if KLC1 suppression has gross affects on the cellular composition of the PA6 differentiation cultures incubated for seven weeks, we used Western blotting to survey a panel of species-specific and neuronally enriched markers. Because of limited *shKLC1-2* material we were focused our analysis on the control and *shKLC1-1* lines. Since mouse PA6 feeder cells may linger in hESC differentiation cultures, we first assessed the relative contribution of these feeder cells by comparing levels of “housekeeping” proteins Actin, Glyceraldehyde 3-Phosphate Dehydrogenase (GAPDH) and Superoxide Dismutase (SOD1) in PA6 feeder cells cultured for seven weeks in the presence or absence of control or *shKLC1-1* hESC. Mouse and human SOD1 and GAPDH proteins have different electrophoretic mobilities so the presence or absence of the mouse and human bands can indicate the contribution of mouse PA6 cell derived compared to human protein. The mouse SOD1 and GAPDH bands were easily detectable in PA6 feeder cell lysates, but not in samples derived from PA6 cells cocultured with either control or *shKLC1-1* hESC (Figure 3A), implying to us minimal PA6 mouse cell contamination within the differentiation cultures.

We estimated the relative proportion of neurons and glia in control and *shKLC1-1* seven week PA6 feeder differentiation cultures by assessing relative levels of neuron marker Neuron Specific Enolase (NSE) and glial marker Glial Fibrillary Acidic Protein (GFAP) by Western blot. Since these cultures also likely contained other hESC differentiation progeny, we used the ubiquitously expressed protein Actin as a normalizer. Although on average there appeared to be less relative NSE and GFAP protein in *shKLC1-1* compared to control differentiation cultures, this trend did not reach statistical significance (p = 0.13 and p = 0.10, respectively; Figure 3B–C), indicating that the proportions of neurons and glia within control and *shKLC1-1* PA6 differentiation cultures were not different.

**Figure 4:**
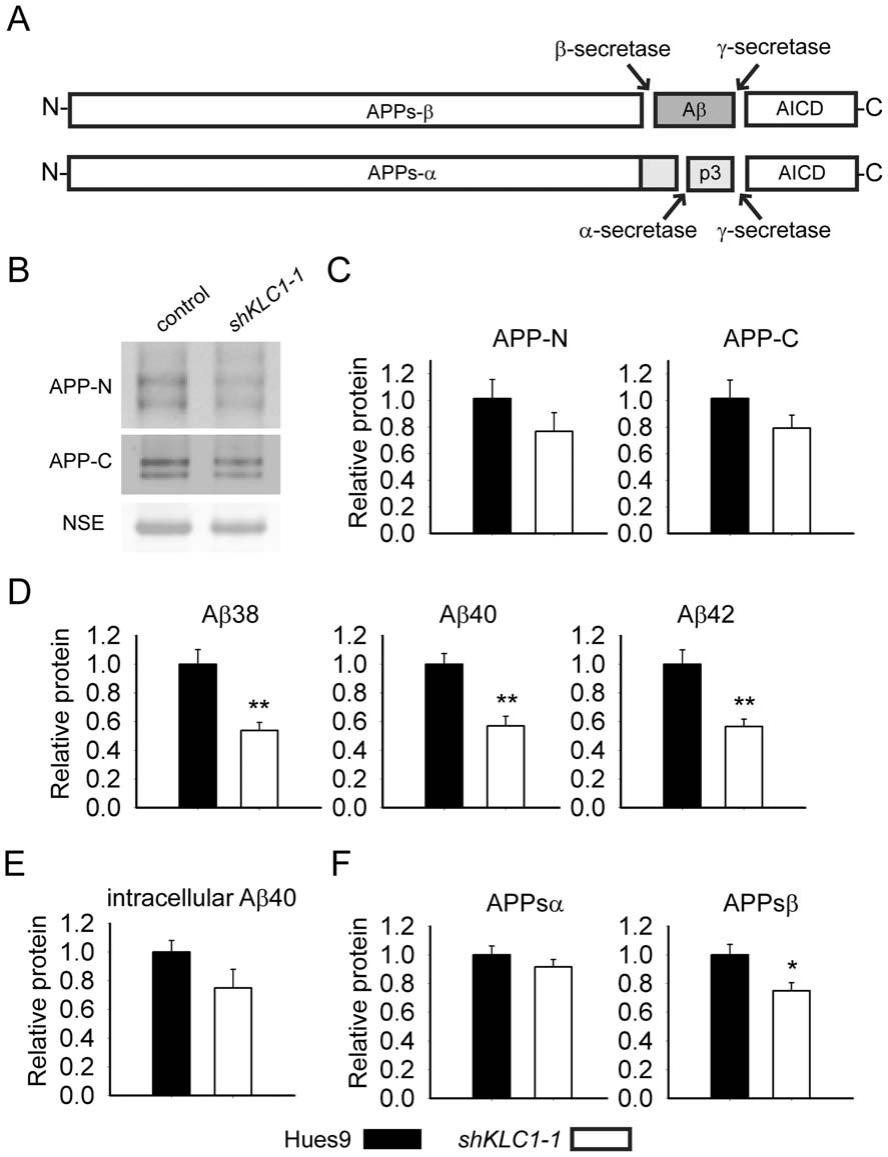
Human neural cultures with reduced KLC1 exhibit altered APP metabolism. (A) APP proteolytic processing by either β-and γ-secretases or α- and γ-secretases produces sAPPβ and Aβ (shaded dark grey) or sAPPα and p3 fragments, respectively. (B–C) PA6 feeder neural differentiation cultures were harvested after seven weeks and equal protein from control and *shKLC1-1* cultures were analyzed using Western blots. (B) Representative immunoblots for full length APP in control and *shKLC1-1* neural differentiation lysates. Results for both amino (APP-N; LN27) and carboxy terminal (APP-C) antibodies are shown. The APP carboxyl terminal cleavage fragments were not reliably detected. (C) Quantification of full length APP levels relative to NSE. (D) Levels of extracellular human Aβ peptides 38, 40 or 42 amino acids in length detected in media conditioned by control or *shKLC1-1* hESC co-cultured with PA6 feeder cells for seven weeks. Human Aβ was not detected from PA6 feeder only cultures. (E) Levels of Triton X-100 soluble intracellular human Aβ-40 in control or *shKLC1-1* PA6 feeder differentiation cultures aged seven weeks. Intracellular Aβ peptides 38 or 42 amino acids long were not detectable. (F) Levels of human extracellular sAPPα and sAPPβ were detected in media conditioned by control or *shKLC1-1* PA6 feeder cocultures aged *in vitro* for seven weeks. Based on n = 6 each line; *p<0.05, **p<0.01 by 2-tailed t-test.

Our imaging data led to us propose that human neuron-like cells produced in PA6 differentiation cultures from hESC with reduced KLC1 may have shorter projections than control cells (Figure 2A right-most panel and Figure S2). To test whether KLC1-suppressed hESC produce neuron-like cells with normal proportions of the microtubule components enriched in neurites, we compared levels of Actin normalized α-Tubulin, β-III-Tubulin, the dendrite marker microtubule-associated protein 2 (MAP2) and axonal markers (pNF; heavy –pNF-H and medium - pNF-M chains) and full length Tau (using both phosphorylation-dependent and -independent antibodies). Interestingly, *shKLC1-1* compared to control differentiation cultures had 25–30% less microtubule subunits α-Tubulin and β-III-Tubulin (an isoform enriched in neurons) and MAP2 (Figure 3B–C). Axonal markers pNF-H and pNF-M were also reduced by 25–35% while Tau was down by >60%, regardless of phosphorylation state (Figure 3B–C). These observations suggest that while pluripotent cells with reduced KLC1 are capable of differentiation to neuron-like progeny, the process is less efficient, producing fewer overall progeny and neurons with shorter projections and less MT-associated cytoskeletal components. Because APP metabolism is linked to AD, it is important to understand how it is regulated in human neurons at endogenous levels. APP associates closely with KLC1 and its axonal transport is Kinesin-1 dependent [20]–[23]. Reduction of full length murine KLC1 in adult mice expressing transgenic human familial AD-associated APP perturbs brain Aβ levels [26]. Aβ is produced by the sequential cleavage of APP by β-secretase and then γ-secretase, while APP cleavage by the α-secretase prevents formation of Aβ peptides (Figure 4A). To assess whether human KLC1 depletion alters APP metabolism in human neural cultures, we measured levels of full length APP and its extracellular metabolites in control or shKLC1-1 hESC PA6 differentiation cultures aged seven weeks. To account for possible differences in the fraction of neurons between cultures we normalized the values to NSE. While levels of full length APP (Figure 4B–C) or soluble intracellular Aβ (Figure 4E) in control compared to shKLC1-1 PA6 feeder cultures were not significantly different, secreted extracellular Aβ levels were substantially reduced in KLC1-suppressed neural cultures (Figure 4D). Regardless of KLC1 levels, 99% of the Aβ40 detected was found in the extracellular fraction. We also tested if PA6 differentiation cultures derived from shKLC1-1 compared to control hESC have similar levels of extracellular sAPPβ or sAPPα fragments. We discovered that KLC1 depletion did not alter levels of sAPPα, but KLC1 depleted neural cultures had less extracellular sAPPβ than control (Figure 4F). These results suggest that KLC1 suppression in human neurons may perturb APPβ, but not APPα cleavage of endogenous APP.

### Neural precursors with reduced KLC1 do not proliferate normally

**Figure 5:**
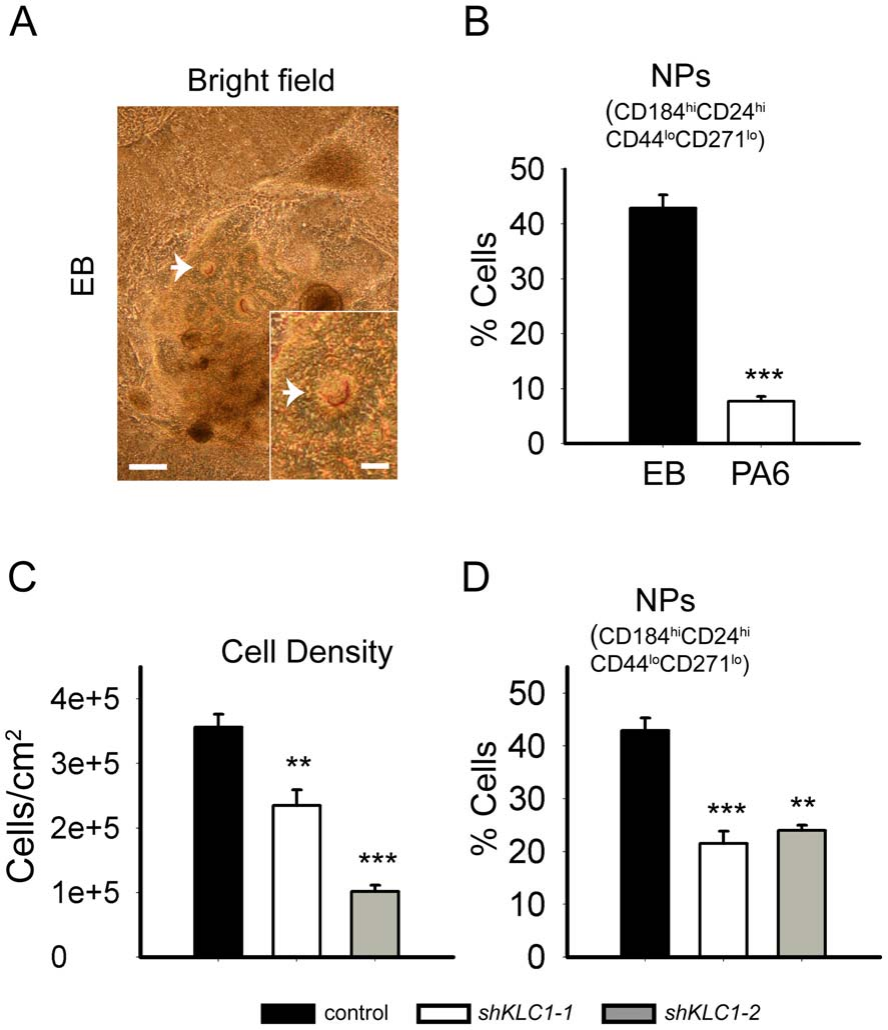
Neural induction cultures made from KLC1-suppressed hESC have reduced cell densities and proportions of NPs. (A–D) Control, hESC were subjected to neural induction conditions for eighteen days using PA6 feeder or EB methods as indicated. (A) Bright field image of control EB neural induction culture (see Figure 2A for image of PA6 differentiation). Arrows point to rosettes. Insets show close-ups of indicated rosettes. Scale bars: 200 µm for main images, 50 µm for insets. (B) Percent of cells in control EB and control PA6 feeder differentiation cultures with CD184^hi^CD24^hi^CD44^lo^CD271^lo^ NP cell surface marker signature (C) Quantification of cell density in EB control, *shKLC1-1* and *shKLC1-2* hESC EB neural induction cultures. EB cultures were dissociated enzymatically and counted using a hemocytometer. (D). Percent of cells within EB control, *shKLC1-1* and *shKLC1-2* hESC differentiation cultures exhibiting CD184^hi^CD24^hi^CD44^lo^CD271^lo^ NP cell surface marker signature after. For (B–C), control n = 9, *shKLC1-1* n = 6, *shKLC1-2* n = 3. For (D), n = 3 each line. **p<0.01, ***p<0.001 by 2-tailed t-test compared to control.

To gain insight into why neuronal cultures derived from hESC with reduced KLC1 are less dense and have reduced microtubule-associated proteins and APP processing, we examined NPs, the cells that divide and give rise to neurons and glia. Flow cytometry is a useful tool for identifying stem cell populations [39] and we have recently developed a flow cytometry-based method to identify and sort out NPs derived from either PA6 feeder differentiation or an alternative neural differentiation method in which nonadherent floating cell clusters, called EBs, are generated and plated on in neuralizing media (Figure S3A) [32]. To determine which method gives the best NP yield, we used both to generate NPs from control hESC. Like the PA6 method, the EB method generated rosette cell clusters (Figure 5A; compare to Figure 2A). Flow cytometric analysis of progeny exhibiting high levels of CD184 and CD24 and low levels of CD271 and CD44, a cell surface signature characteristic of cells which differentiate into neurons and glia (CD184^hi^CD24^hi^CD271^lo^CD44^lo^) [32], revealed that the EB method generated more than four times more CD184^hi^CD24^hi^CD271^lo^CD44^lo^ NPs than the PA6 method (Figure 5B and Figure S3B–C). Therefore, we used the more efficient EB method to ask how reduced KLC1 affects production of CD184^hi^CD24^hi^CD271^lo^CD44^lo^ NPs. Similar to the trend observed with PA6 feeder cultures (Figure 2A), EB neural induction cultures from hESC with reduced KLC1 appeared to have fewer and smaller overall colonies (Figure S4A), suggesting reduced overall cell densities compared to control. We confirmed this observation by quantifying day eighteen control, *shKLC1-1* and *shKLC1-2* neural induction culture cell densities which revealed that KLC1 suppression lowers overall cell densities in a KLC1 dose dependent fashion (Figure 5C). To learn whether KLC1 suppression alters the proportion of NPs within the cultures we used flow cytometry to quantify the percent of control, *shKLC1-1* and *shKLC1-2* PA6 feeder and EB neural induction culture cells with the CD184^hi^CD24^hi^CD271^lo^CD44^lo^ NP signature. PA6 feeder derived shKLC1 neural induction cultures had normal proportions of NPs (Figure S4B). Interestingly, EB neural induction cultures derived from KLC1-suppressed hESC had ∼50% reduced fraction of CD184^hi^CD24^hi^CD271^lo^CD44^lo^ cells (Figure 5D and Figure S3B), suggesting that reduction in KLC1 in hESC can lead to lower NP proportions that could reduce differentiation culture cell densities.

**Figure 6:**
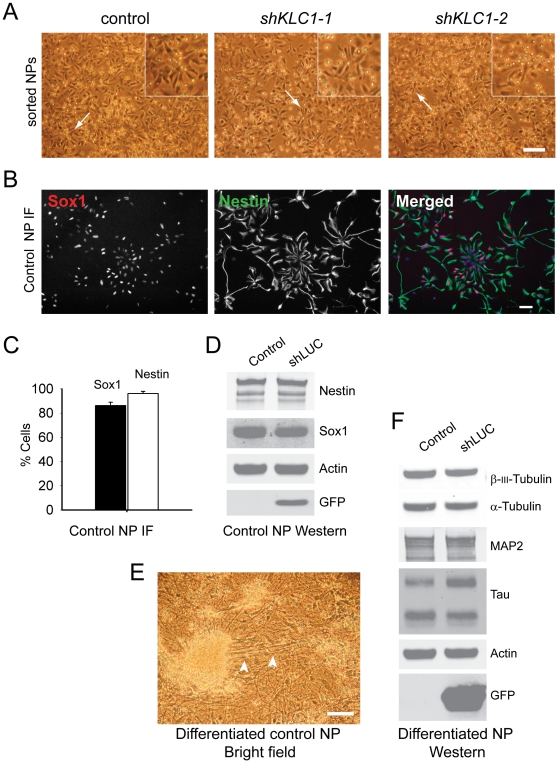
Sorted control but not KLC1-suppressed NPs proliferate and differentiate to neurons. (A) Cells exhibiting the NP cell surface signature were sorted by flow cytometry. Representative images of EB derived NP bright field morphology one day post-sort. Arrows point to individual cells with similar morphology. Scale bar 100 µm. (B) Immunofluorescence for NP intracellular markers Sox1 and Nestin in sorted NP cells from control hESC cultures generated using the EB method. Merged image shows overlay of Sox1 (red), Nestin (green) and DAPI stained nuclei (blue). (C) Quantification of percent of cells ( = DAPI nuclei) positive for Sox1 or Nestin in control derived NP cells. (D) Hues9 derived NPs sorted from EB neural induction cultures were infected with virus containing expression cassettes for a GFP selection marker and shRNA targeting luciferase (shLUC), which is not normally expressed in these cells. Western blotting was used to verify expression of NP markers Nestin and Sox1 in shLUC compared to uninfected control NPs. Also shown are the loading control Actin and the GFP selection marker. (E) Representative bright field image of control hESC derived NPs differentiated for a further 10 weeks. Arrowheads show neurites extending from cell clusters. Scale bar 100 µm. (F) Hues9 derived control or shLUC infected EB derived and flow sorted NPs were differentiated to neurons for five weeks and equal amounts of protein analyzed by Western blotting for α-Tubulin, neuronal markers β-III-Tubulin, MAP2 and Tau as well as an Actin loading control and GFP selection marker.

To address whether KLC1 levels affect NP function, we sorted NPs from EB neural induction cultures derived from hESC with normal or reduced KLC1. Regardless of KLC1 levels, sorted NP cells appeared morphologically similar initially (Figure 6A). However NP cells with reduced KLC1 failed to multiply while NP cells differentiated from control hESC proliferated, expressed NP markers Sox1 and Nestin (Figure 6B–C and Figure 6D, control lane) and differentiated to cultures containing highly polarized cells resembling neurons (Figure 6E) and containing neuronal markers (Figure 6F, control lane). Since sorted NP cells expressing shRNA to KLC1 did not proliferate we were unable to assess their NP marker expression or neuronal differentiation potential by Western blot.

To confirm that KLC1 suppression prevents normal NP propagation in homogeneous NP cultures, we transduced control hESC derived EB NP cells with lentivirus containing a GFP reporter and either shRNA to KLC1 (shKLC1) or a control luciferase shRNA (shLUC) and sorted GFP positive cells (Figure S5). Like uninfected control NP cells, GFP positive shLUC NP cells proliferated, expressed NP markers Sox1 and Nestin (Figure 6D, shLUC lane) and differentiated to cultures containing neuron markers (Figure 6F, shLUC lane). In contrast, GFP sorted shKLC1 lentivirus infected NPs did not proliferate and thus we could not assess their differentiation potential. Taken together we conclude that cellular defects induced by KLC1 suppression can affect differentiation to and impair proliferation of CD184^hi^CD24^hi^CD271^lo^CD44^lo^ NP populations, leading to reduced overall neural differentiation culture densities.

## Discussion

### KLC1 suppression affects microtubule associated proteins

Since KLC1 mutant animals exhibit neural defects, we tested whether reduced KLC1 impairs the differentiation of hESC to neural cells. We found that the neural microtubule-associated markers β-III-Tubulin, MAP2, pNF and Tau are reduced in KLC1 suppressed compared to control cultures. Intriguingly, previous studies suggest that Kinesin-1 may transport Tubulin, thereby regulating cell size and shape [13], [40], [41]. If KLC1 reduction impairs transport of microtubules into neuronal projections, it may cause reductions in cell size, neurite length and levels of microtubule-associated proteins such as MAP2, pNF and Tau. Indeed several studies report reductions in neurite length in KHC or KLC-depleted cultured rodent hippocampal neurons [12]–[14] and in the dendrites of neurons in *C. elegans* and *D. melanogaster* [36], [37]. Reductions in cell size may also partially account for body size reductions observed in Kinesin-1 mutant flies and mice and the reduced white matter tracts observed in the KLC1 mutant mouse [11], [15]–[17]. Alternatively, reduced levels of neural microtubule-associated proteins in the KLC1 suppressed human neural cultures may be due to fewer neurons derived from fewer NPs although neither NSE levels nor the fraction of neuronal (β-III isoform as a fraction of the more widely expressed α-Tubulin) Tubulin were different in neural cultures derived from hESC with normal or KLC1-depleted hESC.

### Effect of KLC1 suppression on APP metabolism in human neural cultures

We found that neural cultures derived from KLC1-reduced hESC have less Aβ compared to control, supporting a functional connection between KLC1 and APP trafficking and/or metabolism. The exact nature of this connection is unknown. However, evidence suggests that Kinesin-1 may transport APP within axons of neurons [21], [26], [42]. The intracellular location of APP is thought to affect its metabolism with α-secretase cleavage likely occurring at the plasma membrane and β-secretase cleavage in endosomal compartments [43]. Once produced, sAPP and Aβ peptides are secreted [44] and Aβ may be degraded by proteases, such as Neprilysin, in the extracellular milieu [45]. Kinesin-1 based axonal transport defects could disrupt any or all of these processes. Our data imply no net effect on extracellular levels of sAPPα in neural cultures derived from hESC with depleted compared to control KLC1 levels, suggesting this cleavage pathway may be normal. However, extracellular levels of β-cleavage pathway products sAPPβ and Aβ are both reduced in the KLC1-suppressed compared to control hESC derived neural cultures, suggesting the APPβ cleavage pathway is disrupted by impaired KLC1. The reduced extracellular Aβ from KLC1-reduced hESC derived human neural cultures agrees with reports of reduced amyloid plaque loads following mechanical disruptions in axonal transport in the perforant pathway of APP transgenic mice [46]. Experiments in better defined human neural cultures will be a first step to understanding the nature of this effect in human neurons.

Work in transgenic mice expressing a familial AD mutant APP suggests that axonal transport perturbations arising from reduced KLC1 function lead to earlier and increased brain Aβ production and plaque deposits [26]. Compared to mice with normal Kinesin-1, animals with reduced KLC1 function also have more Tau in neural tissues [11], [25]. Here we find that human KLC1 depletion in hESC-derived neural cultures reduces endogenous levels of Tau and Aβ. It is unclear why KLC1 disruption in human neural cultures reduces Aβ and Tau while in mouse brain, these proteins are increased. Possible explanations include species specific differences, modes of KLC1 perturbation, neuron type or maturity or differences in production or turnover. Nonetheless, together our data support functional connections between KLC1 and levels of Tau and Aβ.

### A role for the KLC1 subunit of Kinesin-1 in NP maintenance?

Previous studies suggest important functions for Kinesin-1 subunits in the development and maintenance of the nervous system. For example, expression patterns of Kinesin-1 subunits reveal that Kinesin-1A, Kinesin-1B, Kinesin-1C, KLC1 and KLC2 are widely expressed in neural tissues [7]–[10]. In fact, mouse neurons lacking full length Kinesin-1A, Kinesin-1B, Kinesin-1C or KLC1, or Drosophila neurons lacking KHC or KLC, exhibit moderate to severe defects in axonal transport and other neuronal phenotypes [9], [10], [15], [17], [18]. Previous studies in our lab showed that KLC1 reduction in mice leads to altered localization and phosphorylation of Tau while KLC1 heterozygous animals also expressing a human APP transgene exhibit increased brain Aβ, two phenotypes associated with AD [11], [25], [26]. Other work documenting the absence of significant phenotypes caused by loss of Kinesin-1 subunits in virtually all non-neuronal cell types suggest that Kinesin-1, and KLC 1 in particular, does not have “housekeeping” functions [15], [47], [48]. Our finding that KLC1-suppressed hESC do not have growth or other obvious phenotypes is consistent with these previous studies. However, previous findings that Kinesin-1C mutant mice have smaller brains and that KLC1 mutant mice have smaller bodies and reduced white matter compared to wildtype raise the possibility of cell proliferation defects during nervous system development [9], [11], [15]. Therefore, we tested for NP defects in human neural induction cultures with perturbed KLC1. Since over-expression of KLC1 leads to non-physiological cellular aggregation of the protein which is difficult to interpret, we tested the effect of reduced endogenous KLC1 [14], [49]. We found neural induction cultures derived from KLC1-suppressed compared to control hESC have reduced overall cell densities. Our data also show that both NPs sorted from KLC1-suppressed hESC neural induction cultures and sorted control NPs infected with lentivurus coding for shRNA to KLC1 fail to proliferate. These data suggest the hypothesis that KLC1 reduction impairs NP proliferation capacity. Given the neural expression of other Kinesin-1 subunits and the growth retardation defects observed in Kinesin-1A, Kinesin-1B and Kinesin-1C mutant mice, it is possible these other subunits may also have important functions in NP maintenance.

## Supporting Information

Figure S1 **Lentiviral modification, karyotypes and flow cytometric analysis gating strategies for undifferentiated hESC.** (A) Diagram of shRNA constructs used to produce cells with reduced KLC1. The sequences for reverse and forward DNA oligonucleotides with KLC1 exon 2 targeted sequences were: Forward 5′-TGTAATTTGGTGGAGGAGAATTCAAGAGATTCTCCTCCACCAAATTACTTTTTTC-3′ and Reverse 5′-TCGAGAAAAAAGTAATTTGGTGGAGGAGAATCTCTTGAATTCTCCTCCACCAAATTACA -3′ (B) Metaphase chromosome spreads of Hues9 passage 41: 46,XX,inv(9)(p12q13), *shKLC1-1* passage 42: 46,XX,inv(9) and *shKLC1-2* passage 44: 46,XX,inv(9)(p12q13). (C) Hues9 percentile contour plots showing gating strategy to exclude coincident events. (DC) Representative Hues9 percentile contour plots showing gating for pluripotency markers TRA-1-81 and Oct-4. (E) Gating hierarchy for events.
(TIF)

Figure S2 **Morphology of neural cultures derived from control and KLC1 suppressed hESC.** Control, shKLC1-1 and shKLC1-1 hESC were differentiated for seven weeks using the PA6 feeder method. Bright field images show control, *shKLC1-1* and *shKLC1-2* PA6 feeder cocultures at seven weeks since plating. Scale bar = 200 µm.
(TIF)

Figure S3 **Neural induction and neural precursor flow cytometry gating strategies.** (A) Timeline of events for PA6 feeder and EB neural induction cultures. (B) Hues9 control percentile contour plots showing scatter gates for excluding coincident events (top panels) and for both positive (CD184 and CD24) and negative (CD44 and CD271) NP cell markers (bottom panels). Gating hierarchy shown below contour plots. (C) Back-gating of CD184^hi^ CD24^hi^ CD271^lo^ CD44^lo^ population (shown in blue) on CD184 – CD44 & CD271 bivariate dot plots for control, *shKLC1-1* and *shKLC1-2* PA6 feeder and EB neural induction cultures 18 days *in vitro*.
(TIF)

Figure S4 **Additional properties of neural induction cultures.** (A) Control, *shKLC1-1* and *shKLC1-2* hESC were subjected to neural induction conditions for eighteen days using the EB method. Bright field images of neural induction cultures eighteen days *in vitro*. Arrowheads point to rosettes. Insets show close-ups of indicated rosettes. Scale bars: 200 µm for main images, 50 µm for insets. Note the control image is also shown in Figure 6A and is reproduced here for ease in comparison. (B) Percent of cells within PA6 feeder control, *shKLC1-1* and *shKLC1-2* hESC derived neural induction cultures exhibiting CD184^hi^CD24^hi^CD44^lo^CD271^lo^ NP cell surface marker signature.
(TIF)

Figure S5 **Scheme for infection and sorting of NPs with lentivirus expressing shRNA to KLC1 or Luciferase.** (A) A lentiviral vector encoding a GFP expression cassette and either a shRNA targeted to KLC1 (shKLC1) or Luciferase (shLUC) is packaged into virus. (B) Dissociated Hues9 derived NP cells are exposed these virion, (C) GFP positive cells sorted by flow cytometry and (D) plated for expansion.
(TIF)
