# Supplementary figures and images for: Kinesin Light Chain 1 Suppression Impairs Human Embryonic Stem Cell Neural Differentiation and Amyloid Precursor Protein Metabolism
Source: PLoS One. 2012 Jan 17;7(1):e29755. doi: 10.1371/journal.pone.0029755 (PMC3260181; doi:10.1371/journal.pone.0029755)

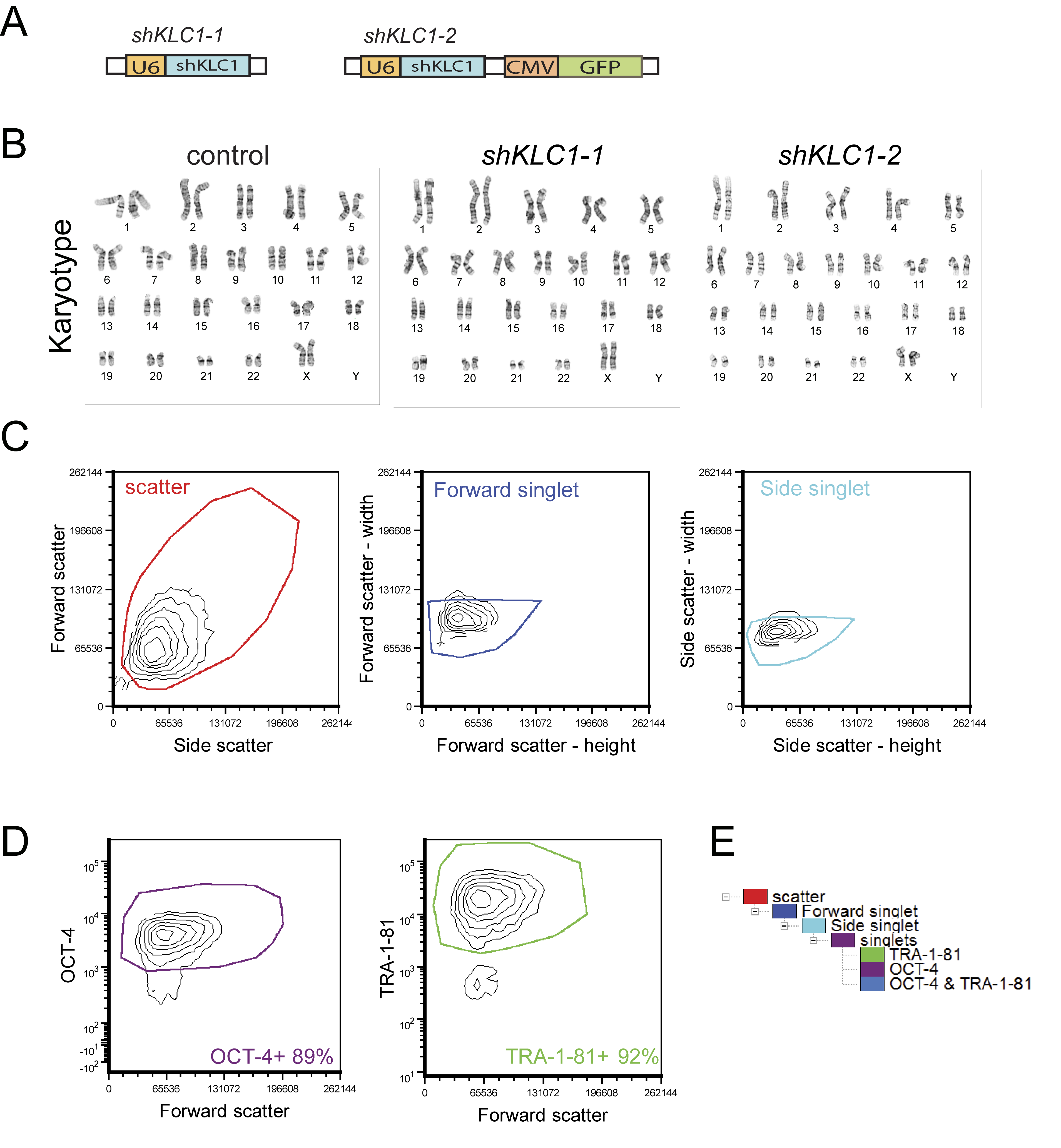

Supplement: Figure S1 — Lentiviral modification, karyotypes and flow cytometric analysis gating strategies for undifferentiated hESC. (A) Diagram of shRNA constructs used to produce cells with reduced KLC1. The sequences for reverse and forward DNA oligonucleotides with KLC1 exon 2 targeted sequences were: Forward 5′-TGTAATTTGGTGGAGGAGAATTCAAGAGATTCTCCTCCACCAAATTACTTTTTTC-3′ and Reverse 5′-TCGAGAAAAAAGTAATTTGGTGGAGGAGAATCTCTTGAATTCTCCTCCACCAAATTACA -3′ (B) Metaphase chromosome spreads of Hues9 passage 41: 46,XX,inv(9)(p12q13), shKLC1-1 passage 42: 46,XX,inv(9) and shKLC1-2 passage 44: 46,XX,inv(9)(p12q13). (C) Hues9 percentile contour plots showing gating strategy to exclude coincident events. (DC) Representative Hues9 percentile contour plots showing gating for pluripotency markers TRA-1-81 and Oct-4. (E) Gating hierarchy for events. (TIF) [file pone.0029755.s001.tif]

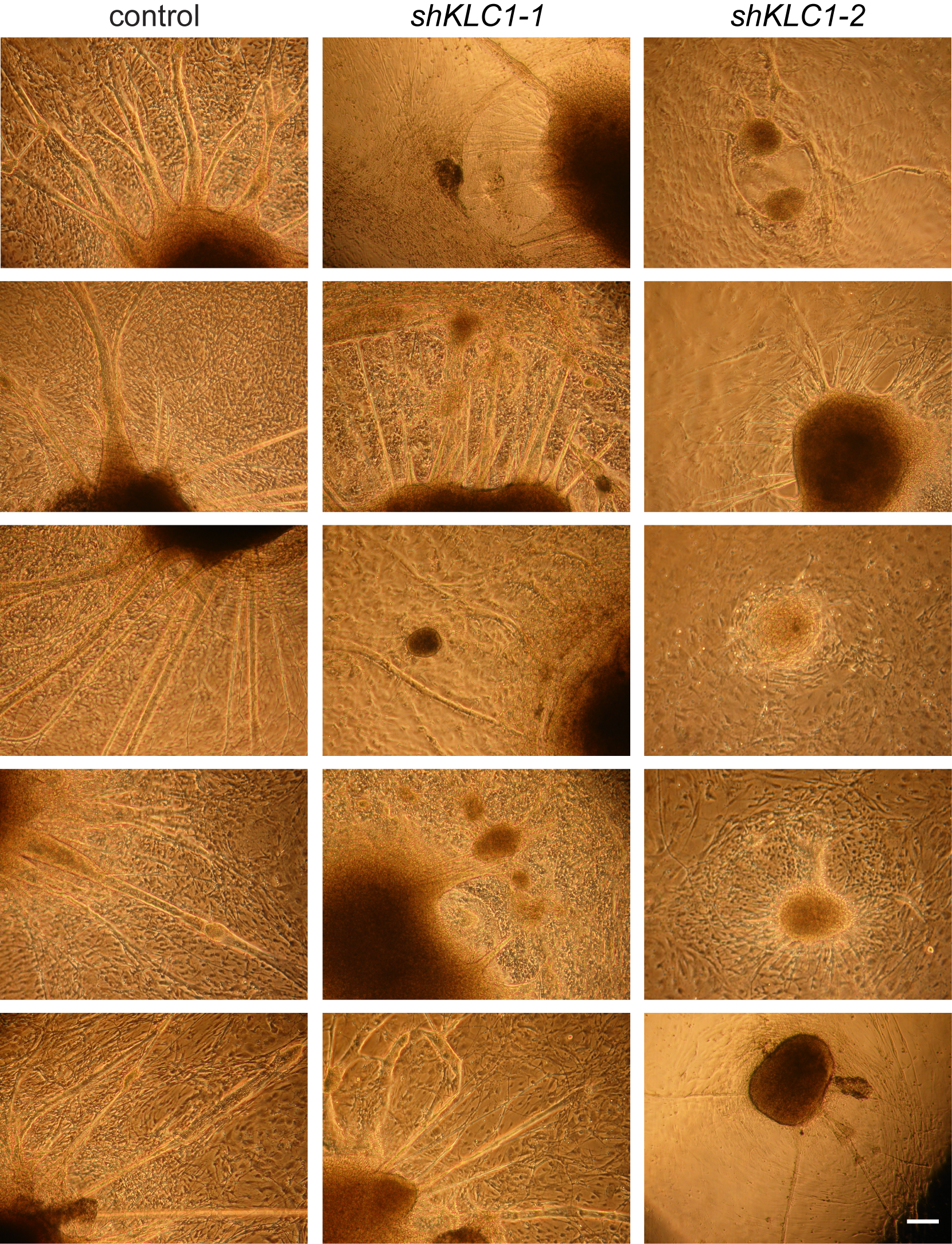

Supplement: Figure S2 — Morphology of neural cultures derived from control and KLC1 suppressed hESC. Control, shKLC1-1 and shKLC1-1 hESC were differentiated for seven weeks using the PA6 feeder method. Bright field images show control, shKLC1-1 and shKLC1-2 PA6 feeder cocultures at seven weeks since plating. Scale bar = 200 µm. (TIF) [file pone.0029755.s002.tif]

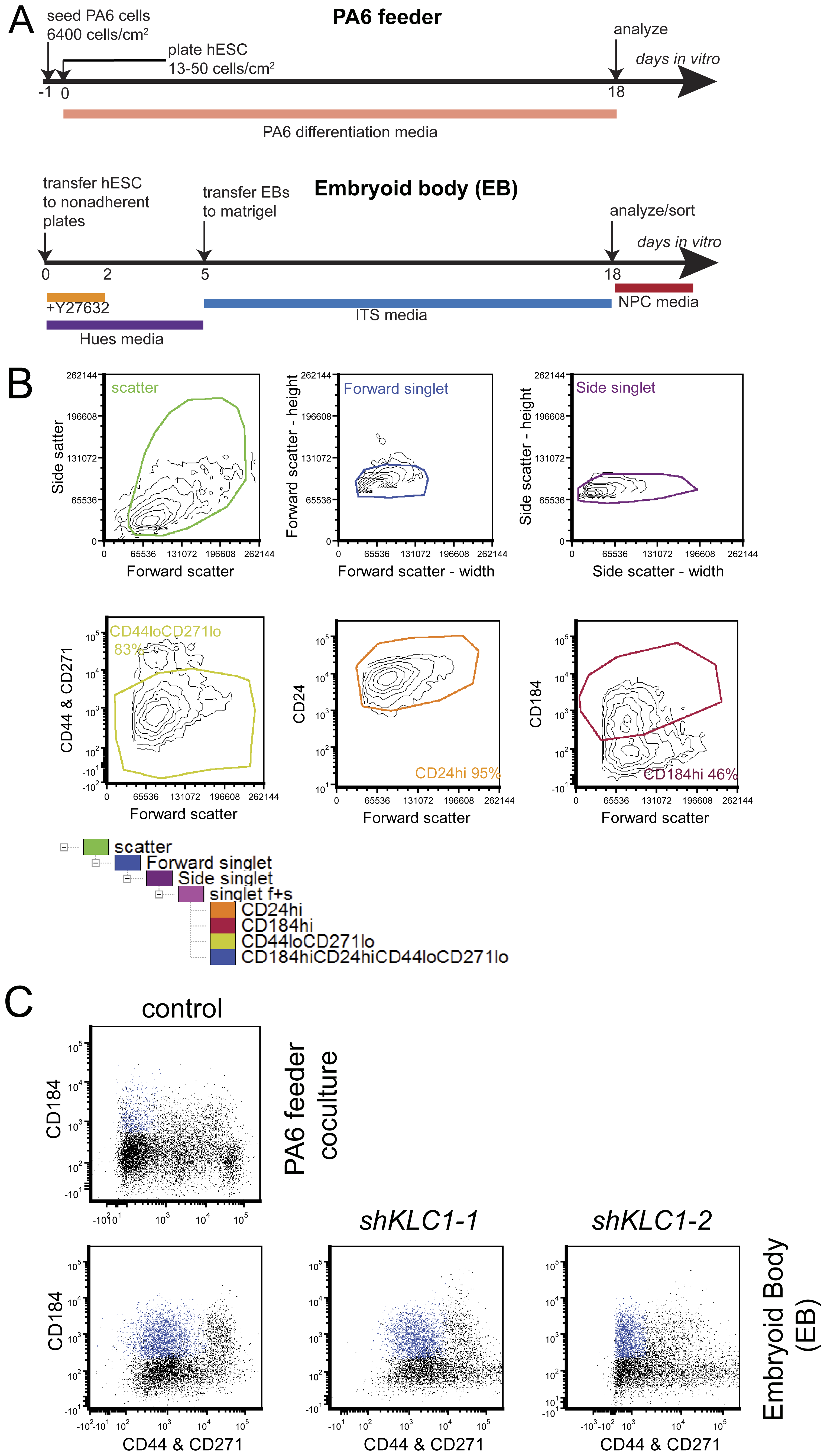

Supplement: Figure S3 — Neural induction and neural precursor flow cytometry gating strategies. (A) Timeline of events for PA6 feeder and EB neural induction cultures. (B) Hues9 control percentile contour plots showing scatter gates for excluding coincident events (top panels) and for both positive (CD184 and CD24) and negative (CD44 and CD271) NP cell markers (bottom panels). Gating hierarchy shown below contour plots. (C) Back-gating of CD184hi CD24hi CD271lo CD44lo population (shown in blue) on CD184 – CD44 & CD271 bivariate dot plots for control, shKLC1-1 and shKLC1-2 PA6 feeder and EB neural induction cultures 18 days in vitro. (TIF) [file pone.0029755.s003.tif]

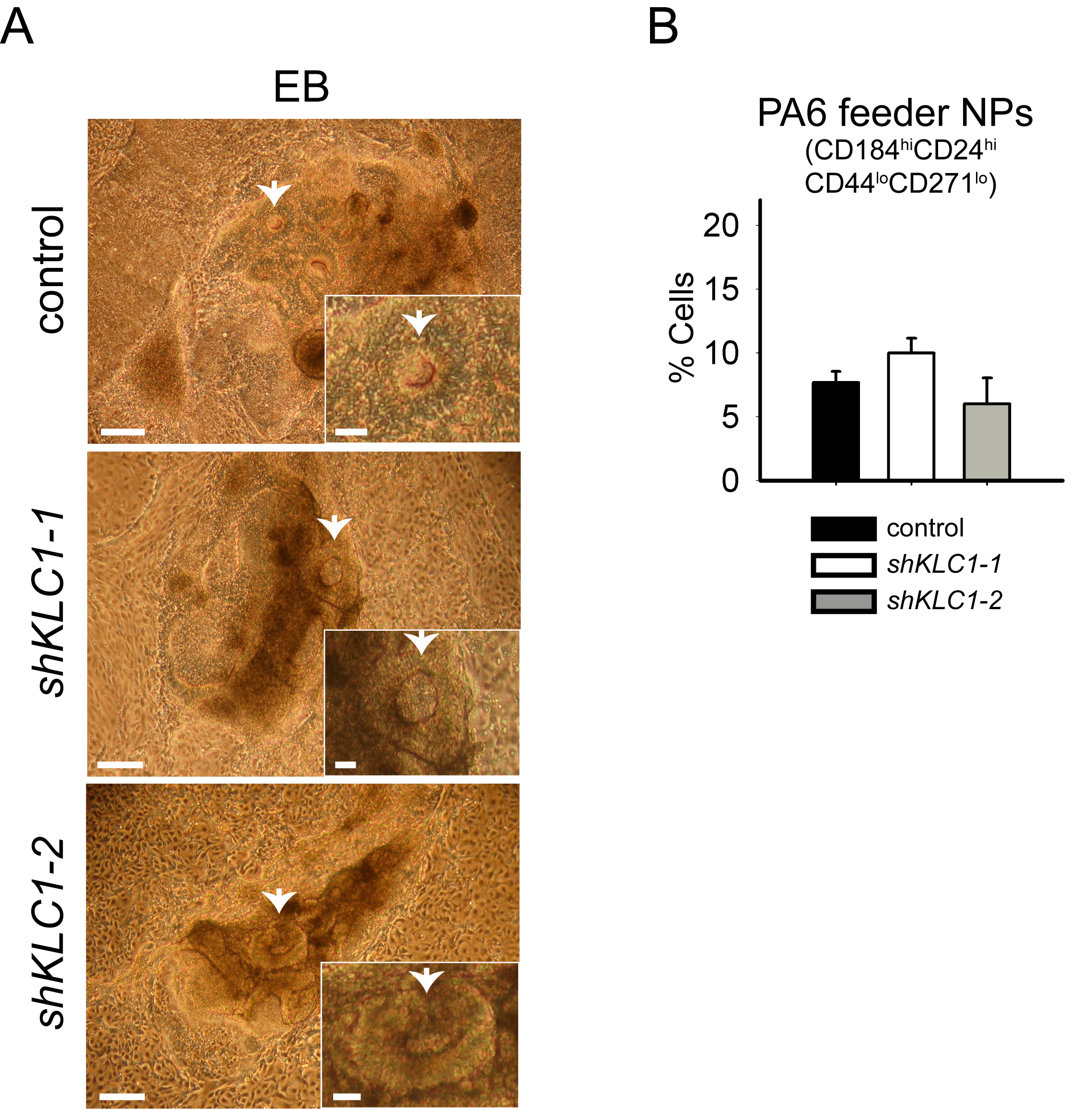

Supplement: Figure S4 — Additional properties of neural induction cultures. (A) Control, shKLC1-1 and shKLC1-2 hESC were subjected to neural induction conditions for eighteen days using the EB method. Bright field images of neural induction cultures eighteen days in vitro. Arrowheads point to rosettes. Insets show close-ups of indicated rosettes. Scale bars: 200 µm for main images, 50 µm for insets. Note the control image is also shown in Figure 6A and is reproduced here for ease in comparison. (B) Percent of cells within PA6 feeder control, shKLC1-1 and shKLC1-2 hESC derived neural induction cultures exhibiting CD184hiCD24hiCD44loCD271lo NP cell surface marker signature. (TIF) [file pone.0029755.s004.tif]

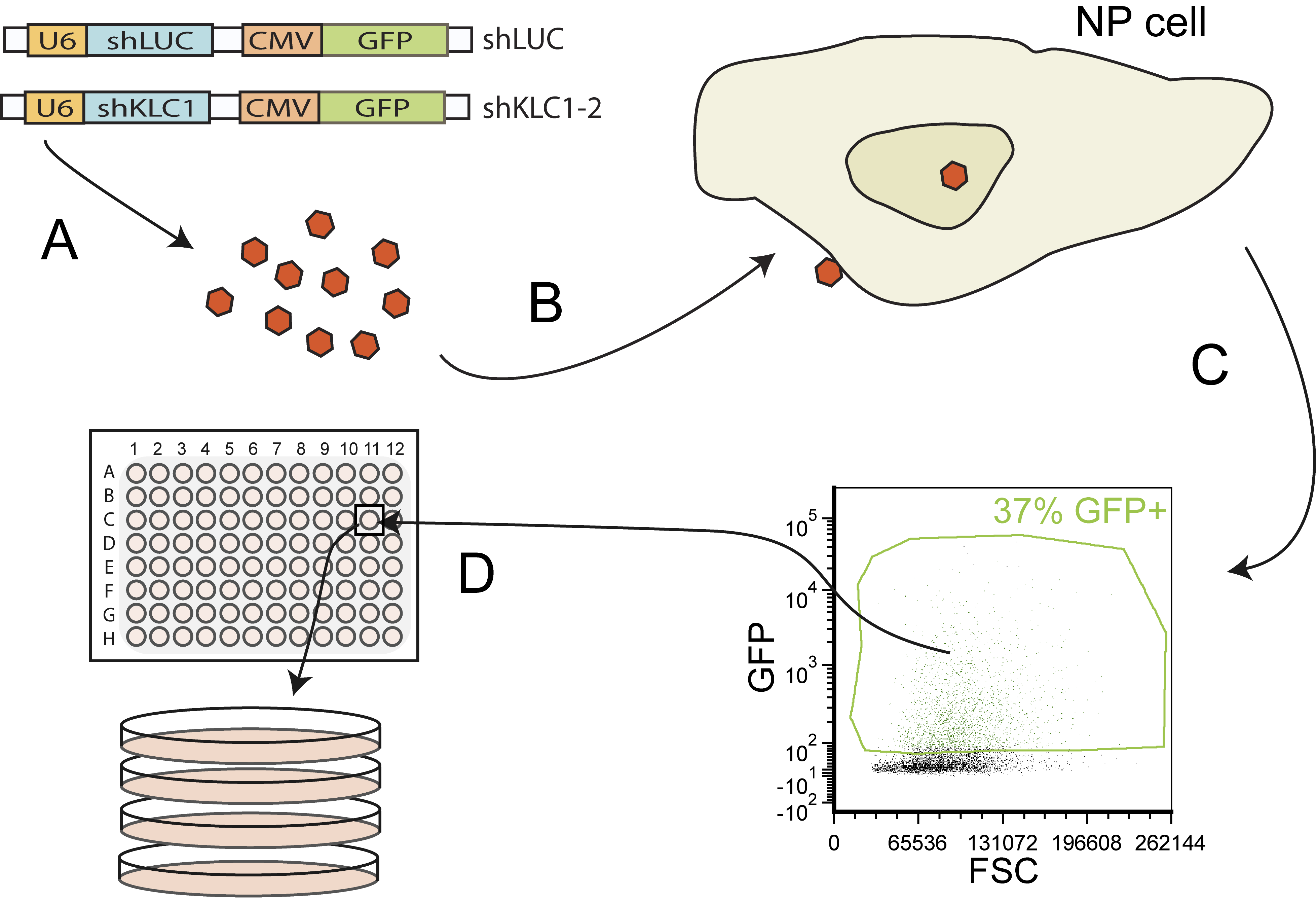

Supplement: Figure S5 — Scheme for infection and sorting of NPs with lentivirus expressing shRNA to KLC1 or Luciferase. (A) A lentiviral vector encoding a GFP expression cassette and either a shRNA targeted to KLC1 (shKLC1) or Luciferase (shLUC) is packaged into virus. (B) Dissociated Hues9 derived NP cells are exposed these virion, (C) GFP positive cells sorted by flow cytometry and (D) plated for expansion. (TIF) [file pone.0029755.s005.tif]
